# DNA sequencing with MspA: Molecular Dynamics simulations reveal free-energy differences between sequencing and non-sequencing mutants

**DOI:** 10.1038/srep12783

**Published:** 2015-08-10

**Authors:** Richard M.A. Manara, E. Jayne Wallace, Syma Khalid

**Affiliations:** 1 Chemistry, Faculty of Natural and Environmental Sciences, University of Southampton, Southampton, SO17 1BJ, United Kingdom; 2 Oxford Nanopore Technologies Ltd, Oxford United Kingdom

## Abstract

MspA has been identified as a promising candidate protein as a component of a nanopore-based DNA-sequencing device. However the wildtype protein must be engineered to incorporate all of the features desirable for an accurate and efficient device. In the present study we have utilized atomistic molecular dynamics to perform umbrella-sampling calculations to calculate the potential of mean force (PMF) profiles for translocation of the four DNA nucleotides through MspA. We show there is an energetic barrier to translocation of individual nucleotides through a mutant that closely resembles the wildtype protein, but not through a mutant engineered for the purpose of sequencing. Crucially we are able to quantify the change in free energy for mutating key residues. Thus providing a quantitative characterisation of the energetic impact of individual amino acid sidechains on nucleotide translocation through the pore of MspA.

Nanopore technology as a basis for next-generation DNA sequencing has received much scientific scrutiny in recent years^1^,^2^,^3^,^4^,^5^,^6^,^7^. The principles of the technique are simple; a protein with a nano-scale aperture is placed in a membrane that separates two chambers containing ionic solution. A voltage is then applied across the chambers, inducing the movement of ions through the pore. The ionic movement is detected as an electrical current. Any charged analyte molecules in the solution will also move through the pore, according to the electric field. In doing so, they cause partial blockage of the current. Similarly, DNA strands can be electrophoretically driven through a nanopore by applying a potential difference. Each of the four DNA bases blocks the current by a different amount, allowing the sequence of the strand to be determined^8^.

Recently the protein *Mycobacterium smegmatis* porin A (MspA) has been shown to be a promising candidate for DNA-sequencing^9^. The geometry of MspA differs from alpha hemolysin from *S. aureus*, which is the other most commonly used protein for nanopore-based sequencing. The constriction region in MspA which has a diameter of 1.2 nm, is only ~1.0 nm in length compared to two constriction regions corresponding to the mouths of the barrel (1.4 nm and 2.6 nm in diameter), which are separated by ~5 nm in alpha hemolysin (Fig. 1). Wildtype MspA must be engineered to incorporate all of the desired properties for application in sequencing. For example it has been shown that a constriction defined by residues D90, D91, and D93 prevents translocation of ssDNA. Mutating these residues to asparagine renders the protein permeable to ssDNA. Exonuclease sequencing is a method in which the DNA is passed through an exonuclease enzyme to separate the strand into individual nucleotides, to better control the speed of translocation^1^. But before considering the use of exonucleases with MspA we may ask what are the energetic differences between the individual nucleotide-protein interactions of the wildtype and mutant protein? Given the smaller size of nucleotides compared to a strand of DNA, are they able to translocate through the wildtype protein? Rational modification of the protein for exonuclease facilitated sequencing, is not possible until these questions are answered.

To this end, here we have employed atomistic molecular dynamics simulations to calculate the free energy differences between the interactions of MspA with an A96R single mutation which we use as a proxy for the wildtype protein (Fig. 2) (this is the mutant for which the X-ray structure has been determined) and the D90N, D91N, D93N, D118R, mutant (henceforth referred to as the M2 mutant) protein with the four DNA nucleotides. The M2 mutant also contains the residues D134 or E139 in reported experimental studies; these residues are omitted in our model due to truncation of the protein. We also note that A96R mutation is not present in the M2 mutant. Specifically, umbrella sampling calculations and WHAM analysis have been used to construct potential of mean force profiles. We have previously used a similar approach to study DNA base and phosphate group permeation through alpha hemolysin^10^.

## Results and Discussion

**Figure 3:**
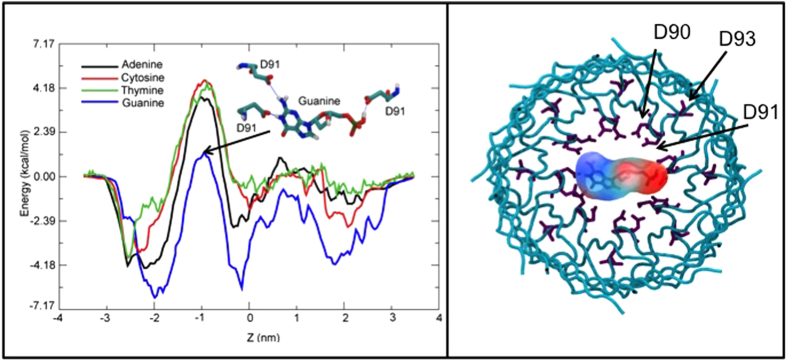
The PMF profiles for the four nucleotides through the A96R mutant, with guanine shown forming hydrogen bonds with the D91 residues in the constriction region (left). Guanine nucleotide in the constriction region at z = 1 nm, with D90, D91 and D93 shown in purple (right).

**Figure 4:**
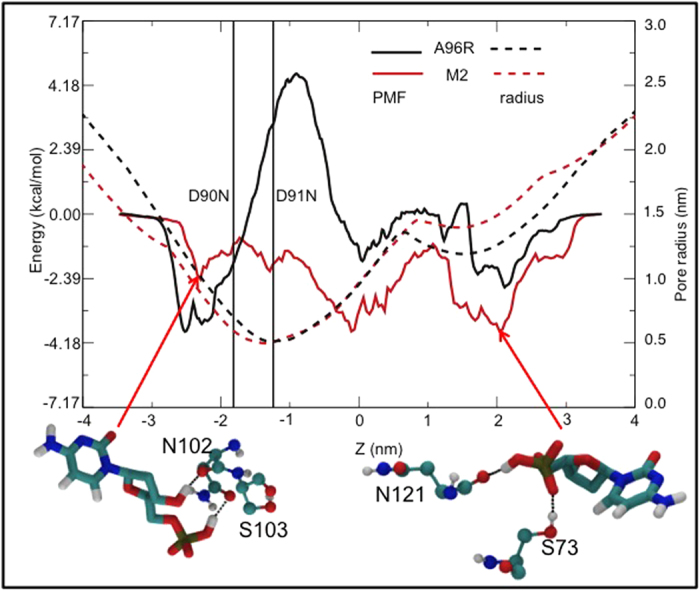
PMF profiles of cytosine monophosphate within the A96R and M2 mutants. Molecular interactions that give rise to two energy wells either side of the central constriction are shown.

Potential of mean force (PMF) profiles reveal several intriguing features (Fig. 3). The profiles for all four nucleotides through the A96R mutant reveal a large energetic barrier corresponding to the constriction defined by residues D90 and D91. The differences between the PMFs for adenine, cytosine and thymine nucleotides are mostly within thermal error. In contrast the barrier for the guanine nucleotide is lower by approximately 3.6 kcal.mol^−1^. The stabilization of guanine is likely a consequence of a combination of steric and electrostatic contributions. The nucleotide is large enough to form 3 hydrogen bonds simultaneously with the sidechains of residues D90 and D91 when lying with its long axis perpendicular to the principal axis of the protein; indeed it adopts this orientation for the majority of the simulation (Fig. 3). In contrast, the other three nucleotides preferentially lie with their long axis aligned parallel to the principal axis of the protein presumably to avoid the phosphate group pointing towards the acidic sidechains of D90 and D91, but in doing so also reduce the scope for hydrogen-bonding. The dimensions of the pore in the A96R barrel were measured (Fig. 4). The pore radius profile along the z coordinate is overlayed on the PMF curve shown in Fig. 4. The peak in the PMF clearly corresponds to the narrowest region of the pore, which has a radius of just ~1.0 nm. Thus we hypothesize that the narrow dimensions of the constriction as well as reduced ability to form hydrogen-bonds are as important in excluding the nucleotides than the charge of the constriction-lining residues.

Having identified the magnitude of the barrier at the constriction in the A96R mutant, we next turned our attention to the M2 mutant. We first focus on cytosine monophosphate. In the M2 mutant, most notably the large barrier calculated at the constriction of the wildtype pore is absent (Fig. 4). Thus removal of the charged aspartic acid residues D90, D91 and D93 contributes >−6 kcal.mol^−1^ to the stabilization of nucleotides within the M2 mutant of MspA compared to the A96R mutant. Comparison of the cytosine monophosphate PMF curves corresponding to the two mutants provides a striking illustration of the effect of substitution of D90, D91 and D93 (Fig. 4). The overall shapes of the profiles are similar except for the obvious absence of the energetic barrier in the M2 mutant. At the trans exit of the pore, where z ~ −2.6 nm, we observe an energy well. Here the stabilizing molecular interactions arise from the phosphate group of the nucleotide forming long lasting (>50 ns) hydrogen bonds to the side chains of residues S103 and N102. The ribose hydroxyl group also forms hydrogen bonds to N102, which provides additional stabilization in this region (Fig. 4). At z ~ 2.0 nm, which corresponds to the cis entrance to the pore, hydrogen bonding between the nucleotide phosphate group and either residue S73 or N121 of MspA is observed throughout the duration of the simulation, while the ribose hydroxyl group forms hydrogen bonds to residue S73. Interestingly, in both cases the residues that provide the stabilizing interactions are asparagine and serine.

**Figure 5:**
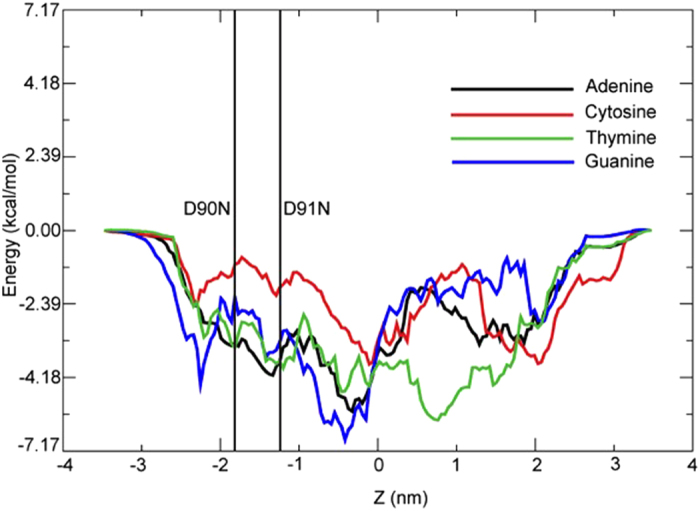
The PMF profiles for the permeation of the four nucleotides through the M2 mutant.

There are no energetic barriers to the translocation of any of the four nucleotides through the M2 mutant (Fig. 5). The adenine, thymine and guanine nucleotides have similar profiles in terms of overall shape, for the region z = −3 to 0 nm, corresponding to the middle of the pore to the trans exit, although the profile for thymine differs from the other two for the remainder of the pore. The profile for cytosine is similar in shape to the other nucleotides in the lower part of the pore, although higher by ~1 kcal.mol^−1^.

**Figure 6:**
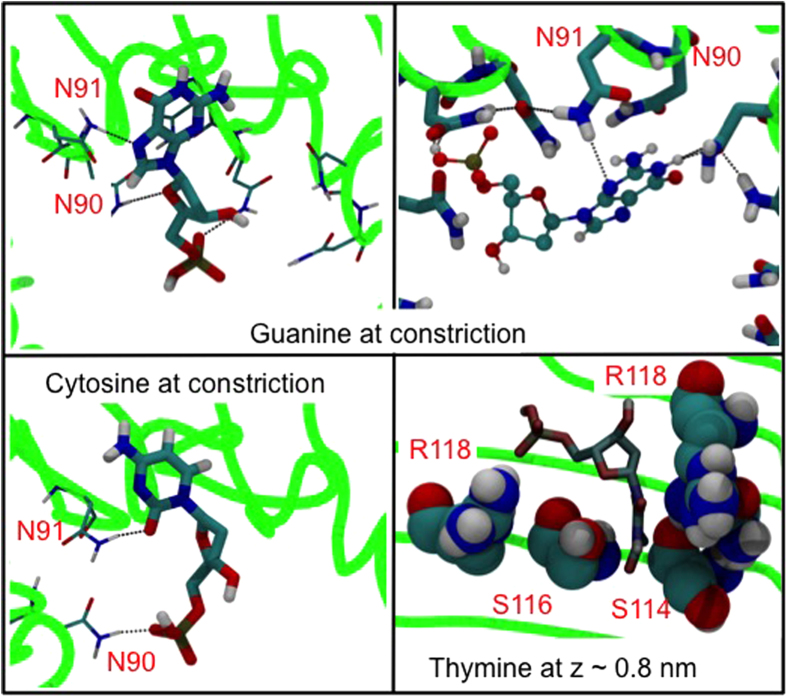
Specific molecular interactions: Guanine forms extended hydrogen bonding networks at the constriction region (top), cytosine can only form, on average 2 hydrogen bonds in the same region (bottom, left) and thymine fits into a pocket formed by S114, S116 and R118 (bottom, right). The green lines depict the protein backbone.

The constriction is widely taken to be the nucleotide discriminating region^9^. Here the energy well for guanine is deeper than for the other nucleotides. In contrast to the A96R mutant, in the M2 mutant, guanine adopts an orientation with its long axis parallel to the principal axis of the pore, for the majority of the simulation. The increased stabilization compared to the other nucleotides is due to its larger size and increased hydrogen bonding capacity. For example, comparison with cytosine, which has the least favorable energy in this region, reveals that guanine forms up to 4 simultaneous hydrogen-bonds with residues 90 and 91 while, cytosine forms on average 2 hydrogen bonds, although up to 3 are possible and are observed intermittently. Furthermore the larger size of guanine means it can form hydrogen bonds that pull asparagine residues closer to each other enabling them to form inter-residue hydrogen bonding networks (Fig. 6). This behavior is not observed with the other nucleotides.

Interestingly, there is a deeper energy well for thymine located at ~0.8 nm–1.0 nm towards the cis exit of the pore. This is absent for the other three nucleotides. A pocket formed by the side chains of residues S114, S116 and R118 provides excellent shape complementarity for thymine at z ~ 0.8 nm (Fig. 6). Interestingly, none of the other nucleotides enter this pocket. This suggests a possible unique recognition site for thymine in this region. Encouragingly previous simulation and experimental studies have shown that triple mutants that incorporate the S116R mutation can give higher currents (sometimes almost twice as high as the wildtype), our simulations predict this mutation is likely to affect thymine monophosphate more so than the other three nucleotides^11^.

It is useful to consider the limitations of the present study, these are largely due to the truncated model of the protein used here, which allow us only to consider the behavior of the nucleotides once they are already inside the pore region. From a sequencing perspective, if the DNA is to be separated into nucleotides, then there must be a practical degree of confidence that nucleotides will not simply diffuse away into the bulk solvent, but will enter the pore. Future studies will include a consideration of this aspect, although the nature of the interactions will necessitate the use of a full atomistic protein and will include two-dimensional PMF calculations.

## Conclusions

Our results reveal the first energetic analysis of the landscape inside MspA as experienced by each of the four DNA nucleotides. We show that the wildtype protein is likely to be as unsuitable for sequencing individual nucleotides, as it is for sequencing strands of DNA, due to contributions from steric clashes, hydrogen-bonding and permanent electrostatic interactions. The range of molecular interactions contributing to the free-energy of permeation render it impossible to predict the subtle differences in the interaction of the four nucleotides with the protein pore by a simple consideration of either the pore/nucleotide geometry, or their respective electrostatic properties. Furthermore, given the emergence of modified nucleotides for gene sequencing, PMF calculations are likely to play an increasingly significant role in aiding the design of nanopores. To this end our study shows that converged PMFs can be achieved for molecules as large as nucleotides on the hundreds of nanoseconds timescale using windows separated by 0.1 nm. Indeed the PMF profiles presented here provide a rational starting point for protein engineering to optimize MspA for application in a DNA sequencing device.

## Computational Methods

Simulations were performed in the GROMACS package^12^ version 4.5.5,^14^,^13^ using the GROMOS 53a6 force-field^14^ and the SPC water model^15^.

We simulate the MspA pore using a similar modeling approach to the one we have previously described for alpha hemolysin^16^,^17^. We truncate the protein to only include the trans-membrane beta-barrel, place the pore in a methane slab then solvate in neutralizing 1 M NaCl solution. The motivation behind implementing such a reduced system is to speed up the simulation time, to enable us to adopt a high throughput computational methodology. We note that the methane particles are subject to weak positional restraints and hence stable in a slab at 310 K, we have previously validated this methodology in our work on alpha hemolysin. Our model pore reduces the total atom count (including water and ions) from hundreds of thousands of atoms, to approximately twenty thousand atoms. Hence, this reduction in the system size leads to a decrease in the required simulation time, rendering our extensive free energy calculations feasible. As the protein is known to be conformationally stable, we do not expect the use of restraints on the protein to influence the PMF, indeed we have previously used a similar approach to calculate PMF profiles for DNA bases through alpha hemolysin^10^. Examples of sampling and convergence testing are provided in figures S1-S5 of the supplementary information. The system was equilibrated in the NPT ensemble for 100 ns, using the Nosé-Hoover thermostat^18^,^19^ and Parrinello-Rahman barostat^20^ to 310 K and 1 Bar prior to the umbrella sampling frame setup. Once the substrates were inserted, the systems were energetically minimized using the steepest descent algorithm. The simulations were run between 150 ns to 250 ns with the first nanosecond discarded for pressure and temperature equilibration. Constraints were used for bond lengths using the LINCS algorithm^21^. Non-bonded interactions were treated with a cut-off of 1.2 nm and the Particle Mesh Ewald method was used for long-range electrostatics. The nucleotides were restrained along the principal axis of the protein, using a force of 1000 kJ.mol^−1^.nm^−1^ remaining free to move laterally within the pore. In the WHAM calculation, the profile was calculated from the pull force, using bins equal to the number of windows. The HOLE package was used to calculate the pore dimensions^22^. Molecular visualization was performed with the VMD graphics package^23^.

## Additional Information

**How to cite this article**: Manara, R. M.A. *et al.* DNA sequencing with MspA: Molecular Dynamics simulations reveal free-energy differences between sequencing and non-sequencing mutants. *Sci. Rep.* **5**, 12783; doi: 10.1038/srep12783 (2015).

## Supplementary Material

Supplementary Information

## Figures and Tables

**Figure 1:**
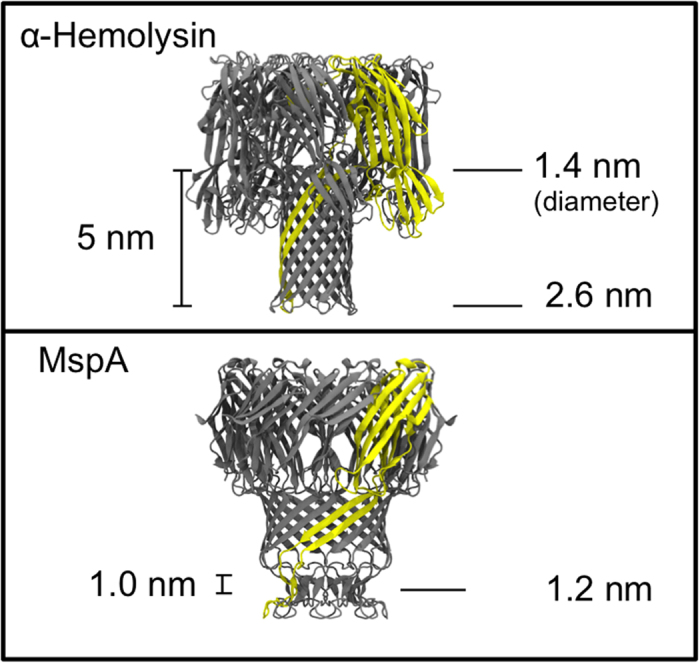
Comparison of the geometries of alpha hemolysin and MspA.

**Figure 2:**
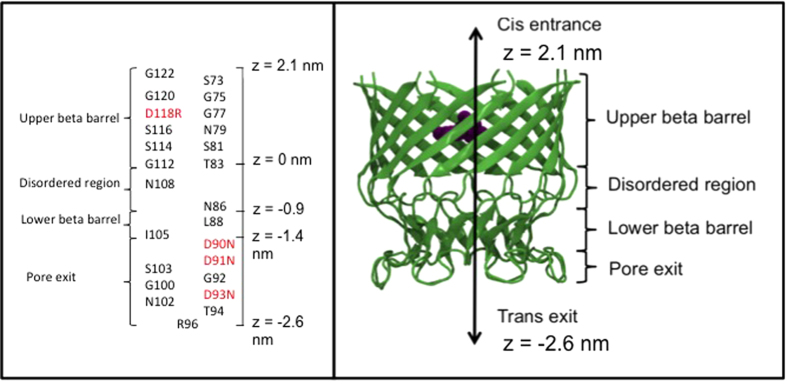
Schematic diagram showing the locations of side chains within the pore (left). Residues in red are mutated. Model MspA aligned along the z dimension, consistent with our umbrella sampling calculations (right).
