# Supplementary material for: DNA sequencing with MspA: Molecular Dynamics simulations reveal free-energy differences between sequencing and non-sequencing mutants
Source: Sci Rep. 2015 Aug 10;5:12783. doi: 10.1038/srep12783 (PMC4530457; doi:10.1038/srep12783)
Supplement: Supplementary Information [file srep12783-s1.pdf]

Supplementary Information

DNA sequencing with MspA: Molecular Dynamics simulations reveal free-energy differences between sequencing and non-sequencing mutants.

Richard M.A. Manara[1], E. Jayne Wallace[2] and Syma Khalid[1]*.

[1]Chemistry, Faculty of Natural and Environmental Sciences, University of Southampton, Southampton, SO17 1BJ, United Kingdom.

[2]Oxford Nanopore Technologies Ltd, Oxford United Kingdom

*Email address for corresponding author: S.Khalid@soton.ac.uk

Validation of methods

It is important to evaluate the convergence of the simulations and to estimate errors associated with the PMF curves in order to be confident about their validity.

Sampling.

A comparison of the sampling in our previous work on αHL and the current simulations of MspA is shown in S1, while S2 shows the orientational freedom of CMP in two umbrella sampling windows.

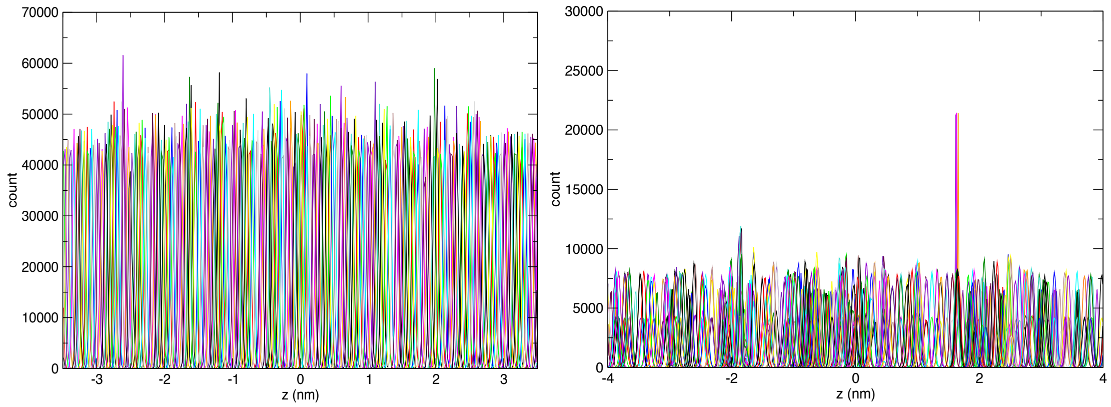

S1: The histograms for cytosine PMF through αHL(left) and CMP through MspA A96R (right). For both sets of simulations, the data show overlap between all of the umbrella sampling windows, indicating that none of the regions along the principal axis of the pore are unsampled. We note however that the MspA histograms are more consistent, with fewer regions of low sampling compared to the αHL histograms.

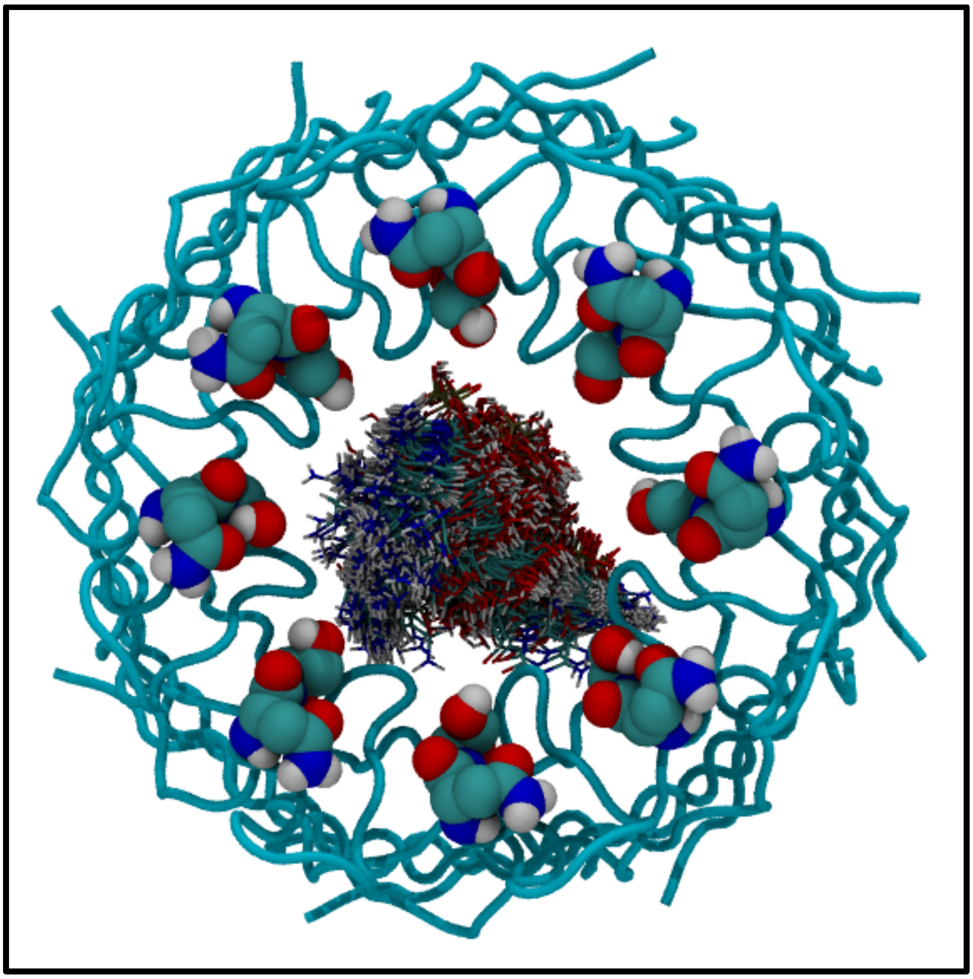

S2: Movement of cytosine monophosphate in the region z = ~-2.6 nm, in which we observed binding for ~ 50 ns. Combining data from two windows in this region shows good coverage of the homo-octamer.

Convergence Testing.

Initially in order to work out the ideal simulation length via block analysis, a PMF was performed with 250 ns per window. This result is shown in S3 for the PMFs produced from the first 25, 50, 75, 100, 150 and 250 ns.

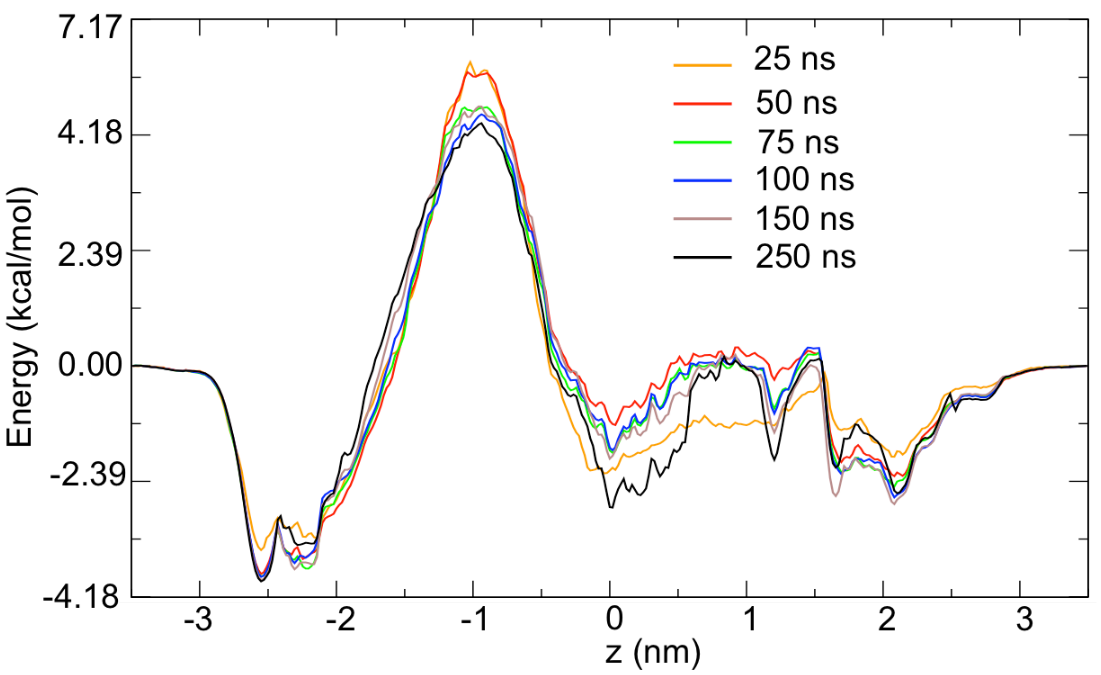

S3: From inspection of the block analysis, we can see that the 25 and 50 ns blocks are not yet converged, as the energies are varying drastically from longer simulations, especially around z=-1.25 nm and z=1 nm. In contrast the 250 ns block appears to sample peaks and troughs not seen by the other simulations e.g. z=-2 nm and z=-0.6. Therefore, based on the block analysis, the remaining simulations were run for 75 ns, as this is the shortest simulation length that samples fully, whilst not getting trapped in conformations.

The bootstrapped profiles were calculated using the Bayesian bootstrap method implemented in g_wham. S4 shows the average profile and standard deviation of 500 bootstrapped profiles for CMP.

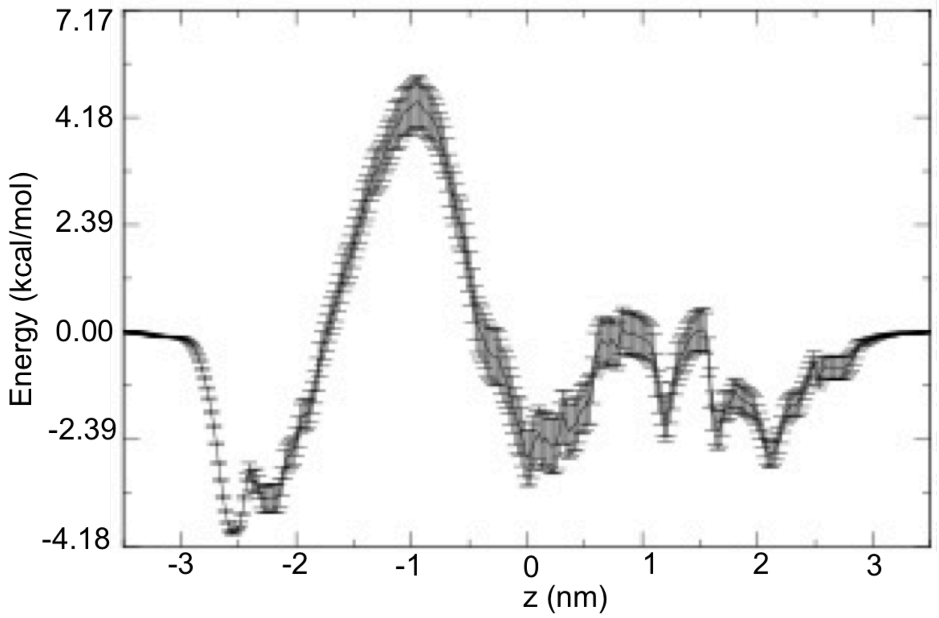

S4: The average of 500 bootstraps for CMP. There are negligible errors in the bulk water and a maximum of 0.64 kcal.mol^{-1} in the protein, as thermal error is 0.61 kcal.mol^{-1} at 310 K this was deemed acceptable as the values compare favorably with our previously published work.

The autocorrelation times were calculated using the GROMACS utility, g_wham, from the pull forces used to construct the PMFs. In S5 we show this data for CMP.

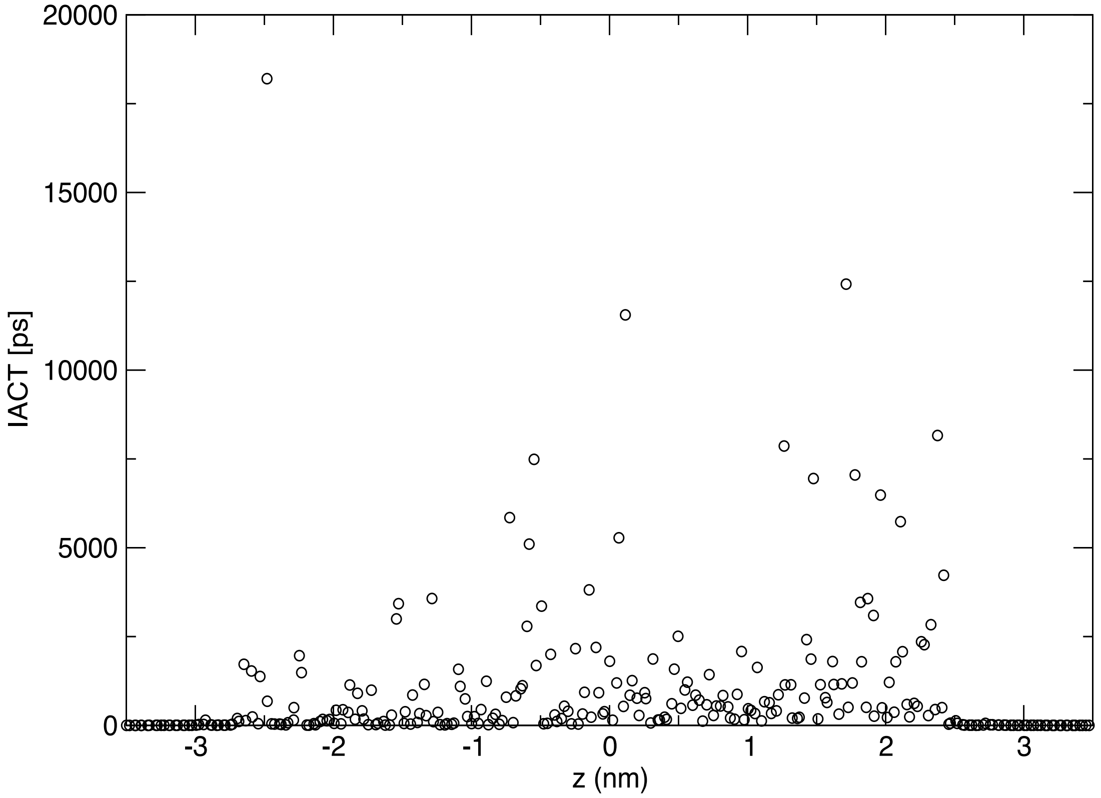

S5: The autocorrelation times are small (generally less than 10 ns) in comparison to the simulation length (250 ns). In only 3 frames is the autocorrelation time above 10 ns.

Reference:

Manara, R. A., Guy, A. T., Wallace, E. J. & Khalid, S. Free-energy calculations reveal the subtle differences in the interactions of DNA bases with alpha-hemolysin. *Journal of Chemical Theory and Computation* (in press).
